# Cli-fi videos can increase charitable donations: experimental evidence from the United Kingdom

**DOI:** 10.3389/fpsyg.2023.1176077

**Published:** 2024-01-19

**Authors:** Ganga Shreedhar, Anandita Sabherwal, Ricardo Maldonado

**Affiliations:** ^1^Department of Psychological and Behavioural Sciences, London School of Economics and Political Science, London, United Kingdom; ^2^Grantham Research Institute on Climate Change and the Environment, London School of Economics and Political Science, London, United Kingdom; ^3^Cine70, Lima, Peru

**Keywords:** climate communications, climate fiction, charitable donations, emotions, videos, experiment

## Abstract

Recent research has begun to investigate if climate fiction, or cli-fi, can increase people’s support for pro-climate initiatives. Emerging evidence focuses on whether cli-fi stories affect people’s self-reported emotions, attitudes, and intentions. Few studies, however, examine the effect of such stories on revealed behavior, and whether the cli-fi story medium, i.e., whether stories are presented in text, audio, or audio-visual format, matters. We investigate the causal effect of cli-fi stories, and the medium through which they are communicated (textual, audio, or audio-visual) on self-reported support for climate policy, individual and collective action intentions, and a revealed measure of charitable donations. In a pre-registered online experiment (*n* = 1,085 UK adults), participants were randomly assigned to one of 5 conditions – to read scientific information about climate change (scientific information condition), read a story unrelated to the environment (control), read a cli-fi story in which a protagonist took intentional pro-environmental actions (fiction text), listen to the same cli-fi story in audio format (fiction audio), or watch an animation of the cli-fi story (fiction video). When comparing the fiction-text, fact-text, and control conditions, we found that cli-fi stories are not always more effective than alternative climate communications: participants in the fact-text condition reported higher support for climate policies, and intentions of taking individual environmental actions, and negative feelings of sadness, disappointment, and guilt, compared to the text-based control and cli-fi text condition. When comparing the cli-fi media format, we found that cli-fi videos were most effective in increasing pro-environmental charitable donations in an incentivized choice task, and self-reported feelings of happiness, hope, and inspiration. The findings show that scientific information about the climate and climate-fiction have an important place in the climate communications toolkit and can offer distinct pathways to enhance support for policy and behavioral change. Communicators seeking to inspire individual pro-environmental actions can consider telling cli-fi stories in video, which may be more compelling. And communicators seeking to enhance public support for societal changes, via climate policies, may benefit from disseminating scientific information about climate change.

## 1 Introduction

Global emissions need to fall by 45% from 2010 levels by 2030 to reach the aspirational limit of 1.5°C set by the 2015 Paris climate agreement. Yet, the latest United Nations Framework Convention on Climate Change report shows that they are likely to increase by more than 10% (UNFCCC, 2022). To achieve rapid emissions reductions, it is widely accepted that both public support for climate policies and behavior change are crucial (Gravert and Shreedhar, 2022; Nature Climate Change, 2022). Public support for climate policies can help induce more structural changes at the local and national levels: by giving a mandate for the expansion of renewable energy and implementation of carbon taxes and eco-labels for example. Similarly, individual, and collective actions in people’s everyday lives can complement and even help drive structural changes: by increasing demand for residential renewable energy and donating to eco-movements that help hold politicians to account, for example.

Given the urgent need to scale individual and societal level action to address climate change, scientists and advocates have increasingly questioned how to make climate communication more effective (Corner and Groves, 2014; Markowitz et al., 2014; Howarth et al., 2020). A frequent and explicit goal of effective climate communications is to grow support for policies and persuade people to change their behaviors, apart from increasing awareness about climate change science *per se* (Markowitz et al., 2014). Yet people encounter diverse types of climate communication in their everyday lives, from more conventional articles disseminating the latest scientific information, to more recently, creative forms of climate communications including literary fiction and poetry. While efforts to tackle environmental issues through creative communications is by no means new, climate change fiction or “cli-fi” has exploded as an important and distinct genre during the last decade (Johns-Putra, 2016; Schneider-Mayerson, 2018).

Alongside the growing popularity of cli-fi, there are also changes in how people consume stories. Climate communications content is increasingly presented using audio (e.g., radio and podcasts) and audio-visual (e.g., short films and documentaries) formats, apart from conventional textual formats (e.g., news articles and stories; Boykoff, 2019). Shifts in climate communication mode have accompanied shifts in reading practices. There has been a decline in reading rates in the United Kingdom for example, and one estimate suggests that in England, roughly 31 and 46% of adults and young people (aged 16 to 24) do not read in their free time. To reach audiences more effectively, therefore, climate communicators frequently make strategic choices between whether to use audio-visual or text media to tell stories. The choice of media format can arguably inform, motivate and capture the imagination of the public in distinct ways (Corner and Groves, 2014; Howarth et al., 2020). Indeed, it is now well understood that “effective climate change communication involves more than simply presenting the scientific information about climate science in a clearer or more concise way” (Corner and Groves, 2014). Despite this, however, the impact of presenting cli-fi in different media formats on people’s preferences for policies and action is unclear.

The aim of this article is to examine how cli-fi impacts public support for climate policy and action. We ask two research questions. First, is scientific information about climate change more effective at increasing support for climate action than fiction? Second, what is the most effective medium to communicate climate fiction? To answer these questions, we used a pre-registered randomized controlled online experiment embedded in a survey in the United Kingdom (*N* = 1,085 adults). To examine if cli-fi is more persuasive than scientific information, we compared text-based communication content (i.e., scientific information vs. fictional story vs. control story). To examine which format of communicating cli-fi was most effective, we compared the same cli-fi story presented using three different formats: textual, audio, or audio-visual formats. We examined effects on both self-reported support for climate policy, individual and collective action intentions, and a revealed measure of pro-environmental behavior, through charitable eco-donations. We also explored effects on some potential psychological mechanisms such as emotions, narrative transportation, and environmental imagination.

This study adds causal evidence to the nascent but fast-growing literature on the impacts of cli-fi on attitudes and behavior and the role of different types of communication media in the climate communication toolkit. It is, to our knowledge, the first study to use a pre-registered randomized controlled trial to elicit causal evidence on impacts of scientific vs. creative climate communication on a wide variety of outcomes including climate policy support, behavior, and emotions. Apart from considering effects on individual and collective action intentions, which is the focus of most past studies, we also measure effects on a revealed behavior – charitable donations, elicited through an incentivized experimental task. We summarize some findings from related literature below.

## 2 Related literature and the current study

### 2.1 Cli-fi, and pro-environmental intentions and behavior

Fictional works about climate change, collectively referred to as cli-fi, has been hailed as a new and important genre to engage people with the issue of climate change, and to inspire public action. In contrast to scientific information, cli-fi stories are seen as a form of narrative persuasion. Narrative persuasion is a form of goal-based communication whereby a persuasive message is embedded within a story, wherein identifiable and relatable characters and events unfold over time in a plot (Escalas, 2007; Moyer-Gusé and Dale, 2017; Appel et al., 2019). The goal of narrative persuasion is to engender a narrative congruent change in the audience’s emotions, beliefs, attitudes, or behavior. Studies have noted that compared to non-narrative formats of communication, narratives can be easier to read and comprehend, more emotionally compelling, and transport the reader more effectively int. the narrative world (Green and Brock, 2000; Escalas, 2007; Dahlstrom, 2014; Bullock et al., 2021). Apart from giving readers new information, or framing information in particular ways, they can also prevent counter-arguing, for example through greater identification with the protagonist’s viewpoint (Dahlstrom, 2014). For example, a growing body of research indicates that narratives can be persuasive at shifting health and consumer beliefs, attitudes and intentions in ways that are congruent with the narrative (Dahlstrom, 2014; Braddock and Dillard, 2016; Appel et al., 2019; Van Laer et al., 2019).

There is limited evidence on the causal effect of cli-narratives on behavior. Existing evidence, that largely focuses on evaluating the effect of reading mainstream climate-themed novels or short cli-fi stories, finds small positive effects on attitudes and intentions (Małecki et al., 2016; Schneider-Mayerson, 2018; Schneider-Mayerson et al., 2020; Malecki et al., 2021). In a correlational study surveying cl-fi readers (*n* = 161 adults, United States), Schneider-Mayerson (2018) found that cli-fi reminded environmentally concerned readers of the severity of climate change and impelled them to imagine environmental futures and consider the impact of climate change on human and nonhuman life. Related studies show that reading an abstract from fictional stories of animals abuse (e.g., from Marek Krajewski’s *Władca Liczb* or *The Lord of the Numbers*) increased pro-animal welfare emotions and attitude (*n* = 1833, Poland; Małecki et al., 2016, 2019; Malecki et al., 2021). Another experimental study, Schneider-Mayerson et al. (2020), found significant positive causal effects of two short cli-fi stories on several important beliefs and attitudes about global warming in a US sample who was concerned about climate change (*n* = 1,671, United States; Schneider-Mayerson et al., 2020). These studies find positive effects on self-reported emotions and attitudes, but do not measure impacts on actual behaviors.

Schneider-Mayerson (2018) cautions that reading cli-fi lead readers to associate climate change with intensely negative emotions, which, could even prove counterproductive to efforts at environmental engagement or persuasion. This is especially likely because of the type of stories told. Most cli-fi stories can be categorized as futuristic dystopia (depicting negative or undesirable futures vs. utopian depiction of positive and desirable futures) or postapocalyptic (depicting future created by an apocalyptic event; Johns-Putra, 2016). Similarly, exposure to information about climate change can also increase negative feelings and decrease wellbeing. For example, exposure to the Intergovernmental Panel’s Climate Change (IPCC) special report on 1.5°C global warming was associated with greater perceived threat from climate change and increased climate change concern in a nationally representative Norwegian sample (Ogunbode et al., 2020). Other work shows a positive relationship between climate communications and climate anxiety (Verplanken et al., 2020; Brosch, 2021). Climate and eco-anxiety encompasses “negative” emotions, like fear, worry, anger and hopelessness (Verplanken et al., 2020; Léger-Goodes et al., 2022), but also behavioral symptoms like anxiety and rumination about personal impacts on the planet (Hogg et al., 2021). Higher awareness and media exposure is associated with higher anxiety, arguably because media content typically refers to risks like increasing number of fires around the world and the rising sea levels, the limited progress made in international climate talks, and so on (Boykoff, 2009; O’Neill and Nicholson-Cole, 2009; Boykoff and Pearman, 2019; Chinn et al., 2020). To alleviate the negative eco-emotions, there have been calls to instead tell stories of present-day heroes taking action now (Ghosh, 2017; Holleman, 2019). In an experimental study, Sabherwal and Shreedhar (2022) found that reading a short cli-fi story about a protagonist taking intentional environmental action in the present is more persuasive at changing individual and collective climate action intentions, but that it did not increase charitable donations (*n* = 903, United Kingdom). Thus, current evidence shows that reading cli-fi stories can increase attitudes and intentions, but not necessarily behavior. Furthermore, in these studies, cli-fi stories are presented as text, and effects are typically compared to a short non-cli-fi story, rather than scientific information.

Another line of research examines whether scientific information, for example climate news and documentaries, are more effective than entertainment or fiction (Cooper and Nisbet, 2016; Morris et al., 2019; Wong-Parodi and Feygina, 2021). However, findings are mixed. For instance, in a controlled experiment (*n* = 158, United States), participants reading a personalized cli-fi short story (i.e., about a protagonist called Annie reducing waste) was found to be more effective than reading eco-waste information at promoting revealed pro-environmental behaviors (e.g., recycling, donations); it also increased self-reported narrative transportation (Morris et al., 2019). In another experimental study, however, there was no differences in participant’s self-reported emotions or behaviors (e.g., email sign up, donations) when exposed to textual narratives (e.g., emotional story linking Arctic warming and harm to polar animals or harm to Santa Claus and his reindeer) compared to scientific information (e.g., about Arctic warming; *n* = 438, United States); in fact, the story conditions seemed to have a negative effect on mitigation intentions. They also found that experiencing negative *and* positive emotions increased acceptance of, concern about, and willingness to act on climate change (Wong-Parodi and Feygina, 2021). These studies, which consider textual and video formats of cli-fi and climate related scientific information, find either positive or no effects on revealed behaviors.

Few studies have also examined differences between news, documentary, and entertainment videos. The available evidence suggests that it is likely that videos and audio formats are more effective than textual formats at changing attitudes and behavior (Gross and Levenson, 1995; Moyer-Gusé, 2008; Shreedhar and Mourato, 2019; Cameron et al., 2021). For example, experimental evidence shows that a documentary (Gasland) was more effective than climate news (and entertainment videos) at increasing negative emotions (Study 1, *n* = 132, United States), but not necessarily risk perception or policy support (Cooper and Nisbet, 2016). Other studies on the effect of videos largely examine the effect of films and documentaries, without comparison to scientific information *per se* (Howell, 2014; Shreedhar and Mourato, 2019; Dunn et al., 2020). For example, there is evidence that exposure to climate films like *Age of Stupid* increased pro-climate attitudes and behavioral intentions (Howell, 2014). In a quasi-experimental study, Jacobsen (2011) found *An Inconvenient Truth* had short-terms effects on carbon offset purchases. Similarly, some controlled experiments show that exposure to short films on human-caused wildlife loss or animal cruelty can increase donations, intentions and emotions like outrage (Merchant et al., 2010; Shreedhar and Mourato, 2019). Yet others show that documentaries like *Blue Planet* had limited effects on lifestyle behaviors like plastic use, even if they increased pro-environmental attitudes and intentions (Dunn et al., 2020). Based on the mixed results from existing studies, therefore, the effect of communication mode on different outcomes remains unclear, and there is little evidence comparing the effectiveness of these different modes in the context of cli-fi.

However, the power of imagery and audio-visual media to promote action has been much remarked upon and analyzed (O’Neill et al., 2013). Recent qualitative research also suggests the public are more favorable to visual and storytelling methods: for example, based on evidence from focus group discussions, participants seem to prefer a continuum of media from video, text, to maps evidence to communicate climate change and climate-affected Lyme disease (Cameron et al., 2021). A meta-analysis of studies employing narratives in the health domain finds that audio/video narratives had a small positive but significant effect size, whereas print narratives had a smaller and insignificant effect size, suggesting that audio and visual narratives are more persuasive (Shen et al., 2015). In line with this, recent causal evidence also shows that audio versions of political narratives elicited greater persuasion than textual versions, because of transportation and feelings of being mentally and emotionally involved in the narrative (Riggs and Knobloch-Westerwick, 2022). Taken together, it is likely that the audio-visual forms of creative climate communication are likely to be more persuasive at increasing climate policy support and actions than textual information.

In sum, there is scarce evidence about the effects of cli-fi. Although mixed, existing literature suggests that textual cli-fi stories could potentially raise support for pro-climate change attitudes and intentions both compared to a control story but also factual text. While there is some promising evidence that reading cli-fi can also increase pro-environmental behaviors like recycling and donations (Morris et al., 2019), there is also evidence finding no effects on intentions, donations and policy support (Cooper and Nisbet, 2016; Wong-Parodi and Feygina, 2021; Sabherwal and Shreedhar, 2022). These studies largely examine the effect of textual cli-fi- narratives and scientific information, but do not systematically explore the effect of the medium of communicating cli-fi itself. Moreover, most studies focus on self-reported attitudes and intentions, not actual behavior.

### 2.2 The current study

In the current study, therefore, we address these gaps in the literature in three main ways. First, we examine whether a short textual cli-fi story is more effective than a control story or factual climate information, adapted from the IPCC. Second, we compare the effectiveness of the same short cli-fi story presented either in a textual, audio, or audio-visual format, to examine whether the communication mode matters. Third, we examine effects on both self-reported intentions (to take collective and individual climate action) and a revealed behavior–charitable environmental donations. In addition, we explore potential psychological mechanisms through which cli-fi media formats may have behavioral effects, including positive and negative emotions, narrative transportation, and environmental imagination.

We have two main research questions. First, are textual cli-fi stories more effective than climate related scientific information or a control story? Second, are cli-fi stories more effective when presented in textual, audio- or audio-visual formats? To answer these questions, we run a between-subjects randomized controlled experiment in the United Kingdom as detailed below.

## 3 Materials and methods

### 3.1 Pre-registration and open data

This study received ethical approval from a UK university’s institutional review board. It was pre-registered at the Open Science Foundation web site. The pre-registration is available.^1^ The stimuli, measures, data, and analyses scripts are available.^2^

### 3.2 Participants and sample size

Our final sample comprised 1,085 UK adults recruited via the online survey platform, Prolific, and the study ran from 15 to 16th March in 2022. The sample size was calculated *a-priori* and pre-registered to obtain more than 80% chance of detecting a small-to-medium effect in one-way ANOVAs with 5 groups and t-tests for pairwise comparisons. Of the 1,091 participants who completed our survey, four failed our seriousness check. As attention checks, participants answered two questions about the story or informational appeal they received during the experiment. Two of the remaining 1,087 participants answered both questions incorrectly and were excluded from the analysis. See Table 1 for socio-demographic characteristics of the sample.

**Table 1:** Descriptive statistics of socio-demographic features across conditions.

Variable	Sample M (SD)	Fiction Text M(SD)	Fiction Video M(SD)	Fiction Audio M(SD)	Control M(SD)	Fact M(SD)
Age	40.50 (12.91)	40.46 (13.06)	39.28 (12.46)	41.65 (12.58)	41.02 (13.76)	40.14 (12.67)
Past Pro-Environmental Behavior	0.67 (0.77)	0.74 (0.79)	0.73 (0.79)	0.63 (0.77)	0.66 (0.77)	0.59 (0.72)
Covid worry	3.51 (1.66)	3.78 (1.69)	3.42 (1.69)	3.46 (1.71)	3.45 (1.61)	3.43 (1.58)
Political Ideology	3.44 (0.88)	3.42 (0.88)	3.40 (0.87)	3.49 (0.88)	3.48 (0.93)	3.42 (0.86)
Income	1.94 (1.02)	2.02 (1.05)	1.93 (1.08)	1.93 (0.96)	1.94 (1.05)	1.89 (0.95)
Education	6.31 (2.68)	6.45 (2.56)	6.43 (2.60)	6.21 (2.71)	6.42 (2.76)	6.04 (2.77)
Literacy	5.91 (0.34)	5.92 (0.31)	5.89 (0.42)	5.92 (0.31)	5.86 (0.37)	5.93 (0.27)

*Age was measured in years; past pro-environmental behavior measured whether participants had donated to an environmental charity and/or joined a protest in the past on a scale of 0 (neither) to 2 (both); Covid worry was measured on a Likert scale of 1 (Not at all) to 7 (Very much); Political ideology was measured on a Likert scale of 1 (Very conservative) to 5 (Very liberal); Income was measured on a Likert scale of 1 (£20,000 or less annual income) to 6 (more than £100,000 annual income); Education was measured on a Likert scale of 1 (Less than O level) to 10 (Doctorate or other professional qualification) and Literacy was measured on a Likert scale of 1 (No English language proficiency) to 6 (Native/Bilingual English language proficiency). See text for gender and ethnicity distributions*.

### 3.3 Experimental design and procedure

**Figure 1:**
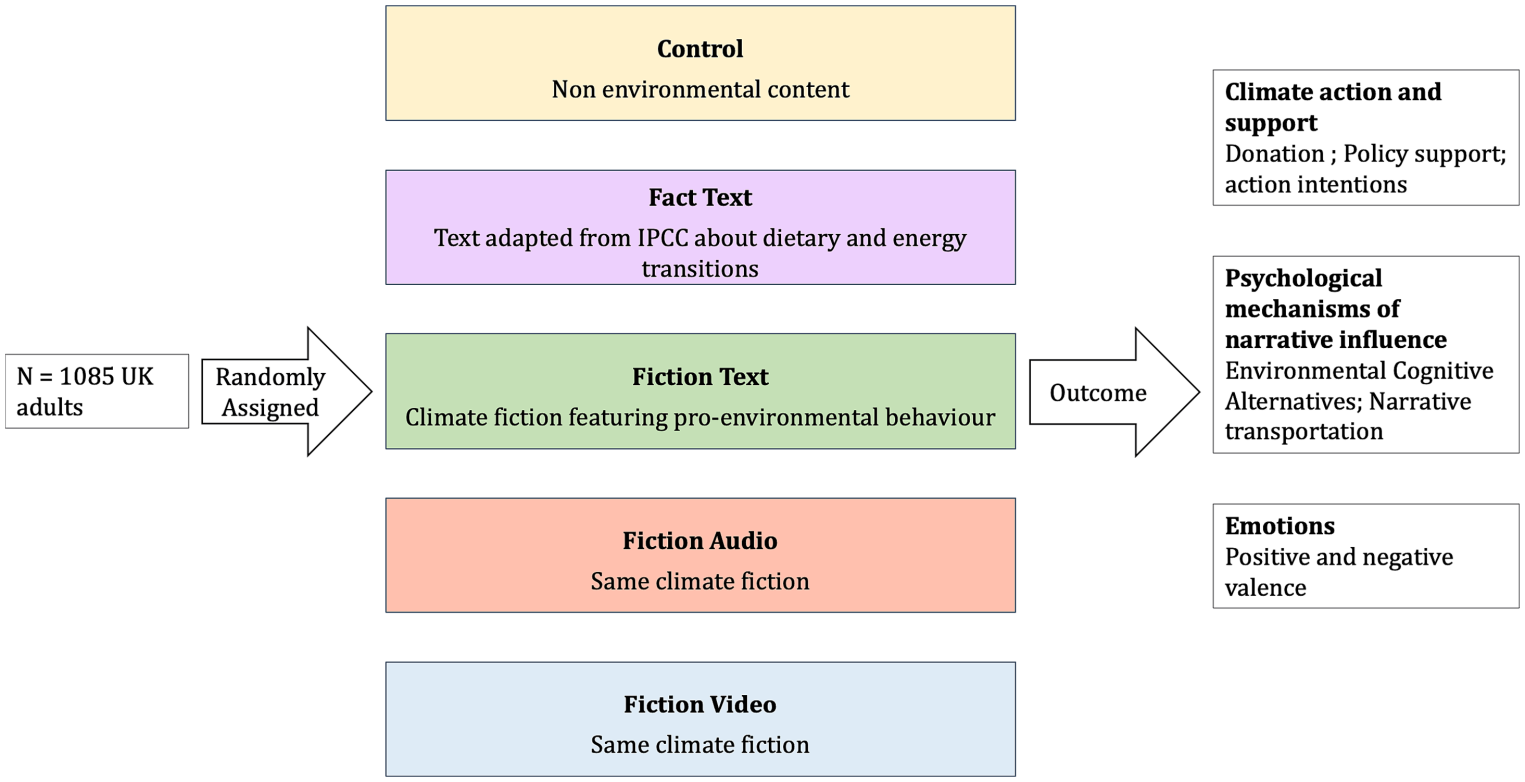
Experimental design.

We used a between-subjects randomized controlled experimental design (see Figure 1), where participants were randomly assigned to one of five conditions after providing informed consent. They either read scientific information adapted from the IPCC report (scientific information condition); or read the intentional environmentalist cli-fi narrative (fiction text condition); or listened to a narration (fiction audio condition); or watched an animation (Fiction-Video condition) or read the control story unrelated to climate change (control condition). The fact, fiction and control stimuli are described below. Next, as attention checks, participants answered two questions asking them to recall information about their respective experimental stimuli.

Participants then reported the extent to which they felt various emotions and were immersed in the stimuli that they had received. Next, participants were asked what proportion of their income from the experiment (if any) they would like to donate to an environmental charity of their choice. They then reported their support for various climate change mitigation policies, their intentions to take collective and individual pro-environmental actions, and their intentions to take collective and individual actions to mitigate climate change. Finally, participants answered questions about their ability to imagine alternative environmental futures (Wright et al., 2020) and provided socio-demographic information. Further details about measures are below. At the end of the survey, participants were thanked for their participation and compensated.

### 3.4 Experimental stimuli

#### 3.4.1 Cli-fi story, scientific information, and control condition stimulus content

Those in the Fact-Text condition read a text adapted from the Intergovernmental Panel on Climate Change’s sixth assessment report (IPCC, 2022). The text informed readers about climate change and discussed behavioral and lifestyle actions (pertaining to dietary changes and energy system transitions) that can aid climate change mitigation and adaptation.

Those in the Fiction-Text, Audio and Video conditions received a story of a day-in-the-life of George, a relatable character who, during his day, makes a pro-climate individual choice [e.g., eating a vegan lunch (instead of a meaty one), and takes collective action (e.g., signing a petition for his organization to target net-zero emissions)]. Both these actions are driven by George’s motive of protecting the environment. This short cli-fi story was written by AS and GS, employed the day-in-the-life narrative structure (which is ecologically valid given its prevalence in both short story formats and social media posts), and was adapted from past research on the role of narratives in mobilizing pro-environmental action (Sabherwal and Shreedhar, 2022).

Those in the control condition also read a story that featured George’s day in the life but did not comprise of environmental actions or any information about the environment and climate change.

Both the Scientific information and Fiction-Text conditions were similar in length (800–900 words) and emphasized the same themes – diet and energy transitions for environmental reasons. The conditions differed in two important ways. Firstly, whereas the scientific information text condition provided scientific information (e.g., “Shifts in dietary choices towards foods with lower emissions and requirements for land, along with reduced food loss and waste, could reduce emissions and increase adaptation options.”), the fiction text condition focused on the protagonist’s mental states (e.g., “He remembered reading that research has shown that emissions from red meat are one of the largest contributors to climate change, and that livestock production also uses-up precious land and water which could be rewilded instead.”). Secondly, unlike the scientific information text condition, the fiction text condition followed a sequential and linear narrative of the protagonist’s day – it featured events from the morning, afternoon and evening of a day in the protagonist’s life (Bullock et al., 2021). Thus, although the length was similar, there were differences in uniformity in reading ease, content, and format, to reflect differences between narrative and non-narrative forms of climate communication.

#### 3.4.2 Media format

For the fiction video condition, we partnered with the animation studio Makaco^3^ to produce a two-dimensional, vectorized animation of the intentional environmentalist narrative (which participants read in the fiction text condition). The audio track included stock library music, sound effects, and a neutral English accent narrator, who was also a climate artist.^4^ Participants in the audio condition heard the same narration as the video. Both conditions were 5 min and 30 s in duration. Complete stimuli are available on OSF.^5^

#### 3.4.3 Measures

Measures of primary interest are listed below. The SI contains a comprehensive list of all measures. All composites were created by aggregating participants’ score on each item.

The main outcomes were climate policy support, individual and collective action intentions, and charitable donations.

##### 3.4.3.1 Donation amount

Participants reported the portion of their income from the experiment they would like to donate to an environmental charity of their choice (*M* = 0.42, *SD* = 0.35) in an incentivized charitable donations task. To avoid confounding effects of their preferred environmental actions, we provided participants a diverse list of environmental charities to choose from. As an adequate incentive, we told participants that we will select 5 participants at random, multiply their income from the experiment and stated donation by 20, and allocate the corresponding amount between participants and their chosen environmental charity.

The donation paradigm was explained to participants via the following set of instructions, **“***You now have a chance to allocate some of your earnings to a charity (Please note that participants were aware that their earnings from the survey were £1. Therefore, they were aware that “allocate some of your earnings” meant allocating some of the £1 they were earning from the survey). After the survey, the research team will select five participants at random, multiply their donation amount by £20 and donate the amount on their behalf. The remaining amount will be sent directly to you with a donation receipt* via *Prolific Academic if you are chosen. Please choose how much you want us to send to you and how much you want us to send to the charity of your choice. If you choose to allocate nothing to the charity, please select ‘£0′ and ‘None of the above’ in the question below*.” This was followed by a slider scale from £0 to £1.

After the study, we selected 5 participants from our participant pool at random, multiplied the amount they had chosen to donate by £20 and donated this to their chosen environmental charity. We reimbursed the rest to them as a bonus on Prolific. For example, if a participant had chosen to allocate “£0.5” to a charity and keep the rest, and they were randomly selected, we donated £0.5*20 = £10 to their chosen charity and gave them a bonus of (£20−£10) £10. This charitable donation paradigm was validated and adapted from previous studies (Carpenter et al., 2008; Charness et al., 2016; Li et al., 2018; Sabherwal and Shreedhar, 2022). We also ensured that participants could understand the pay-out conditions in a pilot experiment, apart from using a pre-validated task. Past evidence shows that the probability of pay-out may affect behavior in similar experimental tasks (Charness et al., 2016); while we cannot rule this possibility out in the current study, the probability of payout and other task instructions was held constant across groups, so that the only difference across groups was the stimulus that they received.

By using this revealed donation measure, we aim to address a growing concern raised in the literature that the excessive reliance of self-reported and non-incentivized donation measures (e.g., by simply asking people to state how much they would donate, if they so could) could lead to over-stating intentions due to hypothetical bias (Murphy et al., 2005; Lange et al., 2023). Although they are seldom present in narrative and climate change communication research, behavioral measures such as the one used above can be important indicators of the down-stream effects of narrative treatments on not only participants’ attitudes but also their actions. Indeed, there is a growing interest in moving beyond solely relying on self-reported outcomes, and also including behavioral methods, to evaluate the effects on climate communications and interventions (Lange, 2023).

##### 3.4.3.2 Policy support

Participants reported their support (1 = Not at all to 7 = Very much; *M* = 4.55, *SD* = 1.59, *a* = 0.92) on 10 climate policies: instituting a meat tax, rewilding, mandating a carbon tax, banning fossil-fuel operated cars, promoting the use of nuclear energy, renovating office buildings for energy efficiency, mandatory carbon offsets for flight tickets, declaring national climate emergency, investing in sustainable aviation fuel research and banning short-distance domestic flights. These items represent policies recommended to meet UK’s target of net-zero emissions (Sabherwal and Shreedhar, 2022).

##### 3.4.3.3 Collective action intentions

Participants reported how likely they would be (1 = Not at all likely to 7 = Extremely likely; *M* = 3.40, *SD* = 1.61, *a* = 0.87) to participate in efforts demanding governmental climate action, sign a net-zero petition and contact government officials to demand climate change mitigating actions. These items were adapted from previous research on collective climate action (Roser-Renouf et al., 2014) and aggregated to create a composite.

##### 3.4.3.4 Individual action intentions

Adapting items from past research (Sabherwal and Shreedhar, 2022), we asked participants to imaging they were hosting a family picnic and answer how likely they would be to talk to their family about making the picnic meatless, go meatless for the picnic, and make the picnic zero-waste (1 = Not at all likely to 7 = Extremely likely; *M* = 3.62, *SD* = 1.67, *a* = 0.80).

##### 3.4.3.5 Narrative transportation

To measure participants’ immersion in the stimuli, we adapted the Transportation Scale short-form (Appel et al., 2015). Participants reported their level of agreement on 7-point Likert scales (1 = Not at all to 7 = Very much) to six statements such as, “I could picture myself in the scene of the events described” (*M* = 4.02, *SD* = 1.21, *a* = 0.85).

##### 3.4.3.6 Environmental cognitive alternatives

Participants’ ability to imagine cognitive alternatives to the environmental status quo was measured using the ECAS scale (Wright et al., 2020). Participants reported their agreement with 10 statements such as, “it is easy to imagine a world where we no longer use fossil fuels” (1 = Strongly disagree to 7 = Strongly agree; *M* = 3.83, *SD* = 1.24, *a* = 0.92).

##### 3.4.3.7 Emotions

Participants rated the extent to which they felt seven emotions – four positive valenced–happy (*M* = 3.32, *SD* = 1.47), surprised (*M* = 2.79, *SD* = 1.43), hopeful (*M* = 3.56, *SD* = 1.54), and inspired (*M* = 3.58, *SD* = 1.58). And three negative valenced–sad (*M* = 2.63, *SD* = 1.64), disappointed (*M* = 2.65, *SD* = 1.63), and guilty (*M* = 2.46, *SD* = 1.46) while reading/watching/listening to the story or message they received as part of the experiment (1 = Not at all to 7 = Very much).

### 3.5 Analytical procedure

As pre-registered, we conducted pairwise comparisons between conditions on dependent variables of interest. To address our first question regarding the effectiveness of climate scientific information and fiction, we conducted an Analysis of Variance, followed by planned pairwise comparisons (T-tests) comparing the fiction text, scientific information text, and control conditions. To address our second research question regarding the most effective medium of communicating climate fiction, we conducted an Analysis of Variance and planned pairwise comparisons (T-tests) comparing the fiction text, fiction audio, fiction video, and control. In the results section, we report all significant analyses of variance and pairwise differences (those pairwise differences not reported were found to be non-significant, i.e., *p* > 0.10).

As such, this analytic approach is consistent with our pre-registration, “The primary analyses will be one-way ANOVAs and planned pairwise contrasts between conditions on the outcome variables mentioned above” (See OSF link for complete pre-registration).^6^

In a deviation from the pre-registration, we conducted two, instead of one analysis of variance for each variable of interest – the first comparing 3 conditions (fiction text, scientific information text, and control), and the second comparing 4 conditions (fiction text, fiction audio, fiction video, and control). This is because each ANOVA corresponded to our two research questions – about the effectiveness of scientific information vs. fiction, and medium (text vs. audio vs. video) of communication, respectively. However, for transparency (and in accordance with our pre-registration), we also conducted one-way Analyses of Variance (ANOVAs) to test the main effect of all 5 treatments (control vs. scientific information text vs. fiction text vs. fiction audio vs. fiction video). These main effects of treatment on variables of interest are reported in the SI (see Supplementary Table 4). Finally, as robustness checks, we also conducted multiple regression analyses to test the effect of each condition, controlling for relevant covariates.

## 4 Results

### 4.1 Sample

The final sample consisted of 1,085 participants, including 536 males and 549 females (50.60% of sample), with the average age of 40.50 (*SD* = 12.91). The sample was 968 White (89.22% of sample), 60 Asian or Asian British (5.53% of sample), and 25 Black, African or Black British participants (2.30% of sample). Of the remaining, 22 identified as belonging to mixed or multiple, and 9 to other ethnicities. The UK population is 51% female, its median age-group is 40 to 59 years. Moreover, 80.6% of the population is White, 7.5% Asian and 3.3% Black (ONS, 2018). Therefore, although our sample consisted of a smaller percentage of Asian and Black participants, overall, it had similar patterns of gender, age, and ethnic distribution as the UK population.

### 4.2 The effect of textual cli-fi narrative vs. scientific information about climate change

#### 4.2.1 Policy support

There was a significant main effect of condition on policy support, *F* (2,643) = 3.15, *p* = 0.04, η_p_^2^ = 0.01. Planned pairwise comparisons found that those in the scientific information text condition (*M* = 4.78, *SD* = 1.49) expressed significantly stronger policy support than those in the control condition (*M* = 4.45, *SD* = 1.64; *t*(419) = 2.15, *p* = 0.03, *d* = 0.21, 95%*CI*[0.02, 0.40]) and those who read the fiction text (*M* = 4.45, *SD* = 1.58; *t*(433) = 2.24, *p* = 0.03, *d* = 0.21, 95%*CI*[0.03, 0.40]).

#### 4.2.2 Individual action intentions

There was also a significant main effect of condition, *F* (2,649) = 3.01, *p* = 0.05, η_p_^2^ = 0.01. Planned pairwise comparisons found that those who read the text in the scientific information condition (*M* = 3.85, *SD* = 1.58) expressed significantly stronger individual action intentions than those in the control (*M* = 3.50, *SD* = 1.70; *t*(425) = 2.21, *p* = 0.03, *d* = 0.21, 95%*CI*[0.02, 0.40]) and fiction text (*M* = 3.52, *SD* = 1.73; *t*(434) = 2.09, *p* = 0.04, *d* = 0.20, 95%*CI*[0.01, 0.39]) conditions.

#### 4.2.3 Donation to environmental charity

There were no main effects of condition on the amount participants chose to donate to an environmental charity and their reported collective climate action intentions (*p* > 0.10).

#### 4.2.4 Additional psychological mechanisms of narrative influence

There were no main effects of condition on participants’ Ability to imagine cognitive alternatives to the environmental status quo (ECAS) and reported narrative transportation (*p* > 0.10).

#### 4.2.5 Emotions

In terms of Happiness, there was a significant main effect of condition, *F* (2,649) = 32.91, *p* < 0.001, η_p_^2^ = 0.09. Those in the scientific information text condition (M = 2.48, SD = 1.22) reported being significantly less happy than those in the fiction text (*M* = 3.36, *SD* = 1.50; *t*(420) = 6.83, *p* < 0.001, *d* = 0.65, 95%*CI*[0.45, 0.85]), and control conditions (*M* = 3.43, *SD* = 1.40; *t*(419) = 7.56, *p* < 0.001, *d* = 0.73, 95%*CI*[0.53, 0.93]). Considering Hope, there was a significant main effect of condition, F (2,649) = 4.17, *p* = 0.02, η_p_^2^ = 0.013. Those in the scientific information text (*M* = 2.28, *SD* = 1.36) condition were significantly less hopeful than those in the fiction text (*M* = 3.61, *SD* = 1.65; *t* (421) = 2.30, *p* = 0.02, *d* = 0.22, 95%*CI*[0.03, 0.41]) condition. There were no significant main effects of condition on the extent to which participants reported feeling inspired and surprised (*p* > 0.10).

There was a significant main effect of condition on Sadness, F (2,649) = 114.9, *p* < 0.001, η_p_^2^ = 0.26. Participants in the scientific information text condition (*M* = 4.22, *SD* = 1.64) reported feeling significantly more sad than those in the control (*M* = 2.41, *SD* = 1.49*; t*(430) = 12.09, *p* < 0.001, *d* = 1.16, 95%*CI*[0.94, 1.38]) and fictional text (*M* = 2.26, *SD* = 1.39*; t*(428) = 13.55, *p* < 0.001, *d* = 1.29, 95%*CI*[1.07, 1.51]) conditions. There was a significant main effect of condition on Guilt, F (2,649) = 97.39, *p* < 0.001, η_p_^2^ = 0.23. Participants in the scientific information text condition (*M* = 3.61, *SD* = 1.56) reported feeling significantly more guilty than those in the control (*M* = 1.95, *SD* = 1.22*; t*(415) = 12.34, *p* < 0.001, *d* = 1.18, 95%*CI*[0.96, 1.40]) and fiction text (*M* = 2.14, *SD* = 1.27*; t*(422) = 10.85, *p* < 0.001, *d* = 1.03, 95%*CI*[0.82, 1.24]) conditions. Finally, there was a significant main effect of condition on Disappointment, F (2,649) = 121.7, *p* < 0.001, η_p_^2^ = 0.27. Participants in the scientific information text condition (*M* = 4.12, *SD* = 1.66) reported feeling significantly more disappointed than those in the control (*M* = 2.25, *SD* = 1.29*; t*(413) = 13.13, *p* < 0.001, *d* = 1.26, 95%*CI*[1.03, 1.48]) and fiction text (*M* = 2.24, *SD* = 1.38*; t*(425) = 12.93, *p* < 0.001, *d* = 1.23, 95%*CI*[1.01, 1.45]) conditions.

Taken together, these findings indicate that compared to climate fiction, the climate scientific information condition increased participants’ policy support, individual action intentions, and negative valenced emotions. Contrastingly, the climate fiction condition increased participants’ positive valenced emotions (compared to the climate scientific information condition).

### 4.3 The effect of cli-fi communication mode

#### 4.3.1 Donation to environmental charity

There was a marginally significant main effect of condition, *F* (3, 860) = 2.17, *p* = 0.09, η_p_^2^ = 0.01. Planned pairwise comparisons found that those in the fiction video condition (*M* = 0.47, *SD* = 0.34) made a significantly larger donation than did those in the control (*M* = 0.39, *SD* = 0.35; *t*(427) = 2.46, *p* = 0.01, *d* = 0.24, 95%*CI*[0.05, 0.43]) condition and marginally significantly larger donation than did those in the fiction audio condition (*M* = 0.41, *SD* = 0.34; *t*(431) = 1.80, *p* = 0.07, *d* = 0.17, 95%*CI*[−0.02, 0.36]). We found no other pairwise differences (*p* > 0.10). Therefore, of all the messages we tested, only the fiction video was able to increase participants’ donations to pro-environmental charities.

#### 4.3.2 Policy supports and action intentions

We found no main effects of condition on participants’ climate policy support, their collective climate action intentions, and their individual climate action intentions (*p* > 0.10).

#### 4.3.3 Additional psychological mechanisms of narrative influence

There was a significant main effect of condition on participants’ Ability to imagine cognitive alternatives to the environmental status quo (ECAS), *F* (3, 860) = 3.89, *p* = 0.01, η_p_^2^ = 0.013. Planned pairwise comparisons showed that those who received the fiction video (*M* = 4.03, *SD* = 1.26) scored significantly higher on the ECAS than did those who received the fiction text (*M* = 3.66, *SD* = 1.24; *t*(437) = 3.11, *p* = 0.002, *d* = 0.30, 95%*CI*[0.11, 0.49]) and fiction audio (*M* = 3.74, *SD* = 1.19; *t*(431) = 2.45, *p* = 0.01, *d* = 0.24, 95%*CI*[0.05, 0.42]) versions of the narrative.

There was no significant main effect of condition (*p* > 0.10) on narrative transportation.

#### 4.3.4 Emotions

The various modes of communication differed in the extent to which they prompted positive valenced emotions. There was a significant main effect of condition on happiness, F (3, 860) = 5.16, *p* = 0.002, η_p_^2^ = 0.02. Participants in the fiction video condition (*M* = 3.86, *SD* = 1.42) reported feeling significantly happier than those in the control (*M* = 3.43, *SD* = 1.40*; t*(430) = 3.17, *p* = 0.002, *d* = 0.31, 95%*CI*[0.11, 0.50]), fiction audio (*M* = 3.51, *SD* = 1.43*; t*(430) = 2.57, *p* = 0.01, *d* = 0.25, 95%*CI*[0.06, 0.44]), and fiction text (*M* = 3.36, *SD* = 1.50*; t*(436) = 3.54, *p* < 0.001, *d* = 0.34, 95%*CI*[0.15, 0.53]) conditions.

Similarly, hope differed significantly across conditions, F (3, 860) = 9.25, *p* < 0.01, η_p_^2^ = 0.03. Participants in the fiction video condition (*M* = 4.01, *SD* = 1.58) reported feeling significantly more hopeful than those in the control (*M* = 3.23, *SD* = 1.43*; t*(428) = 5.40, *p* < 0.001, *d* = 0.52, 95%*CI*[0.32, 0.71]), fiction audio (*M* = 3.68, *SD* = 1.53*; t*(431) = 2.23, *p* = 0.03, *d* = 0.21, 95%*CI*[0.02, 0.40]), and fiction text (*M* = 3.61, *SD* = 1.65*; t*(436) = 2.63, *p* = 0.01, *d* = 0.25, 95%*CI*[0.06, 0.44]) conditions.

Finally, there was a significant main effect of condition, F (3, 860) = 5.36, *p* < =0.001, η_p_^2^ = 0.02. Participants in the fiction video condition (*M* = 3.95, *SD* = 1.55) reported feeling significantly more inspired than those in the control (*M* = 3.37, *SD* = 1.63*; t*(427) = 3.80, *p* < 0.001, *d* = 0.37, 95%*CI*[0.17, 0.56]), fiction audio (*M* = 3.51, *SD* = 1.56*; t*(430) = 2.96, *p* = 0.003, *d* = 0.28, 95%*CI*[0.09, 0.47]), and fictional text conditions (*M* = 3.52, *SD* = 1.68*; t*(434) = 2.78, *p* = 0.01, *d* = 0.27, 95%*CI*[0.08, 0.45]). There was no significant main effect of condition (*p* > 0.10) on the extent to which participants reported feeling surprised.

The modes of communication also differed in the extent to which they prompted negative valenced emotions. There was a marginally significant main effect of condition on sadness, F (3, 860) = 2.54, *p* = 0.06, η_p_^2^ = 0.01. The fiction video condition (*M* = 2.05, *SD =* 1.21) significantly lowered sadness compared to the control (*M* = 2.41, *SD =* 1.49; *t*(406) = 2.68, *p* = 0.01, *d* = 0.26, 95%*CI*[0.07, 0.45]).

There was also a significant main effect of condition on guilt, F (3, 860) = 3.58, *p* = 0.01, η_p_^2^ = 0.01. The fiction video condition (*M* = 2.34, *SD* = 1.34; *t*(429) = 3.11, *p* = 0.002, *d* = 0.30, 95%*CI*[0.11, 0.49]) and fiction audio condition (*M* = 2.25, *SD* = 1.29; *t*(422) = 2.43, *p* = 0.02, *d* = 0.24, 95%*CI*[0.04, 0.43]) significantly increased guilt compared to the control (*M* = 1.95, *SD* = 1.22). However, there was no significant main effect of condition (*p* > 0.10) on the extent to which participants were disappointed.

**Figure 2:**
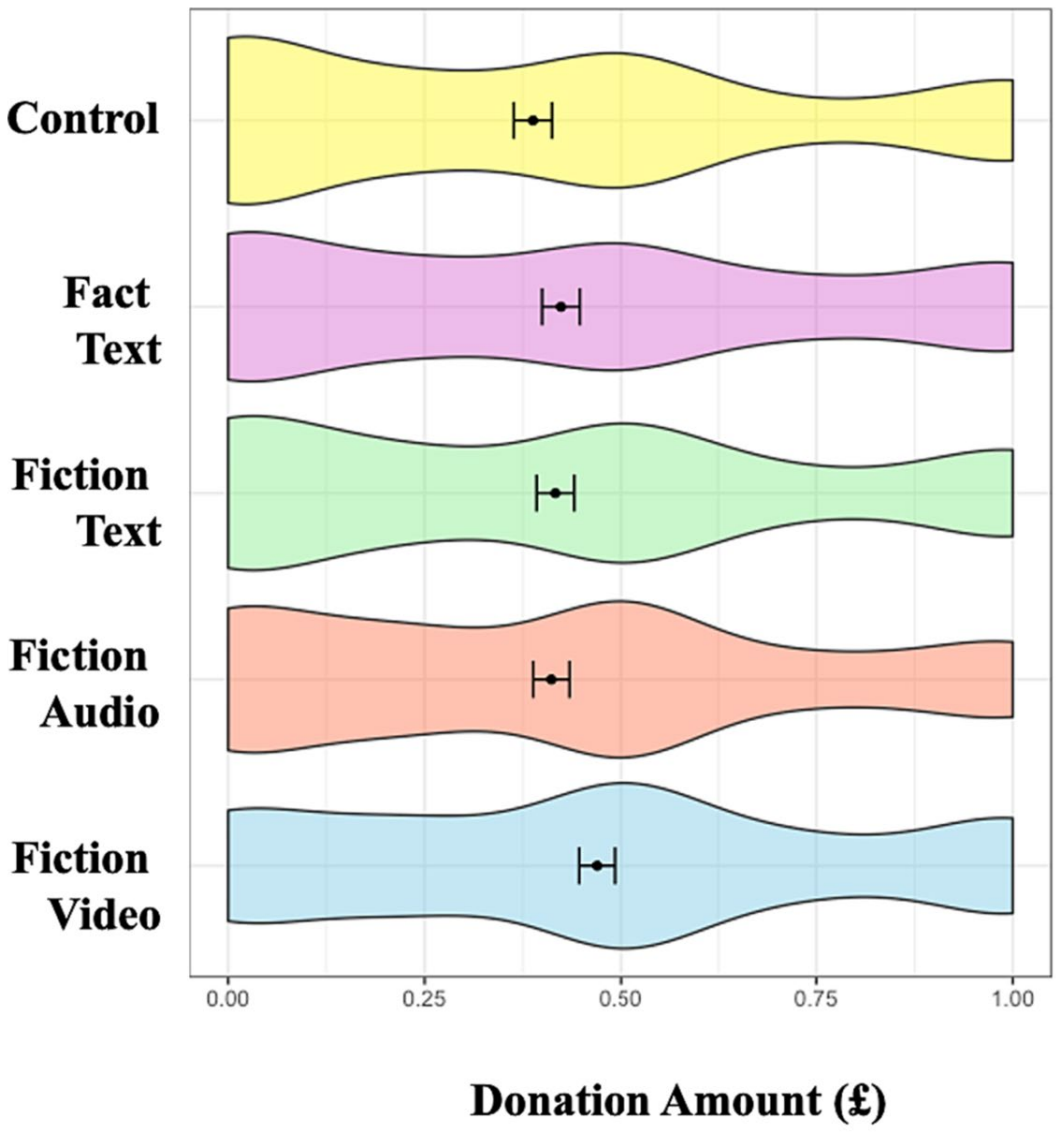
Conditional effects on donation. Error bars represent 95% confidence interval of the means. Donation amount measured in £0 to £1.

**Figure 3:**
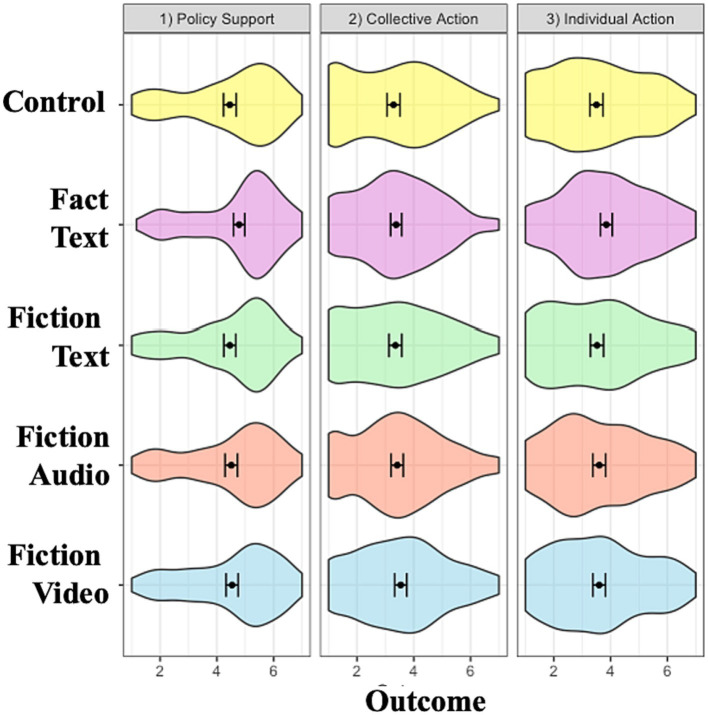
Conditional effects on policy support and action intentions. Error bars represent 95% confidence interval of the means. Policy support, collective action and individual action intentions measured using 10-item, 3-item, and 3-item composites, respectively. All items measured on 7-point Likert scales.

**Figure 4:**
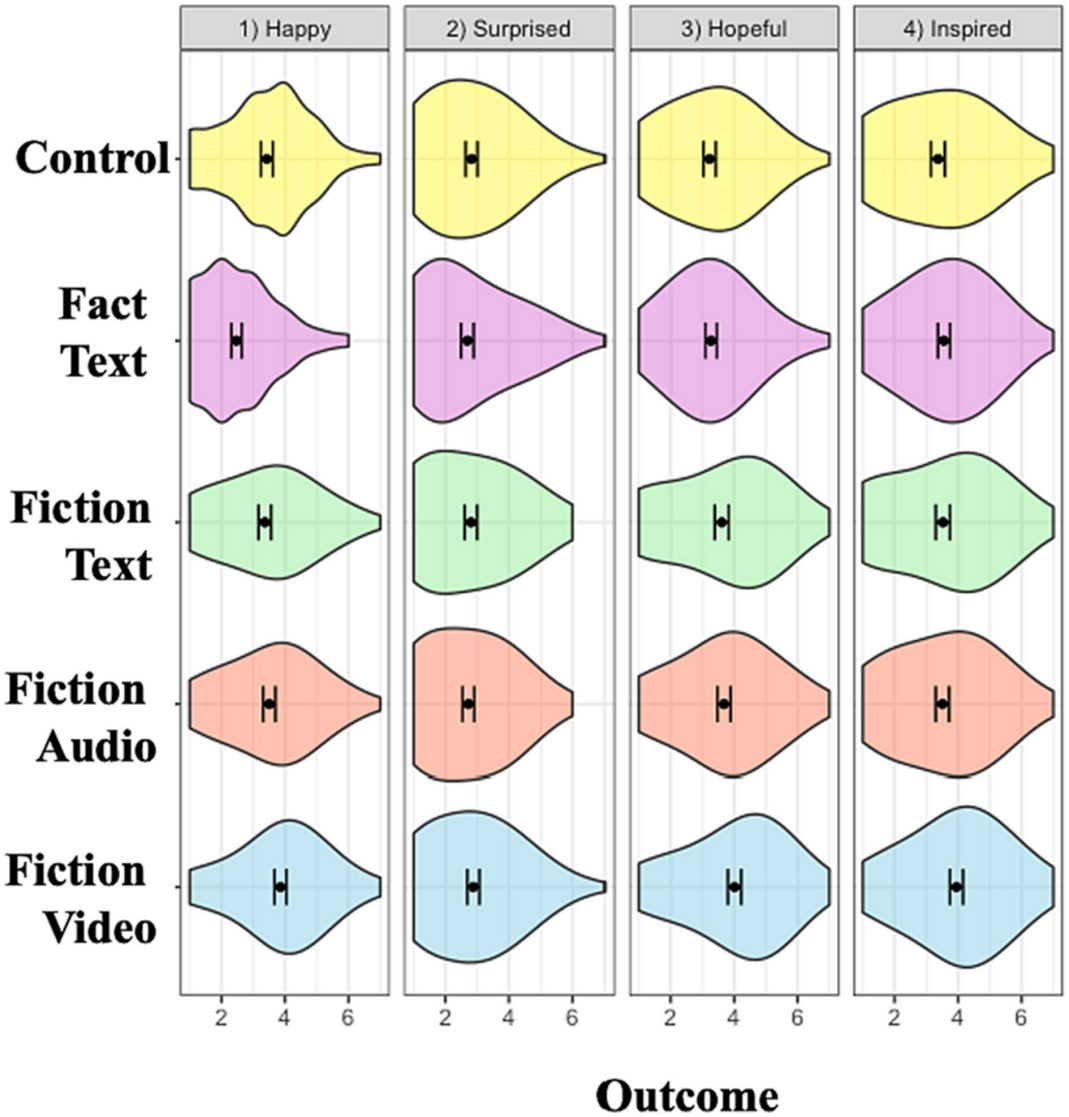
Conditional effects on positive valence emotions. Error bars represent 95% confidence interval of the means. Each emotion measured using a single item on a 7-point Likert scale.

**Figure 5:**
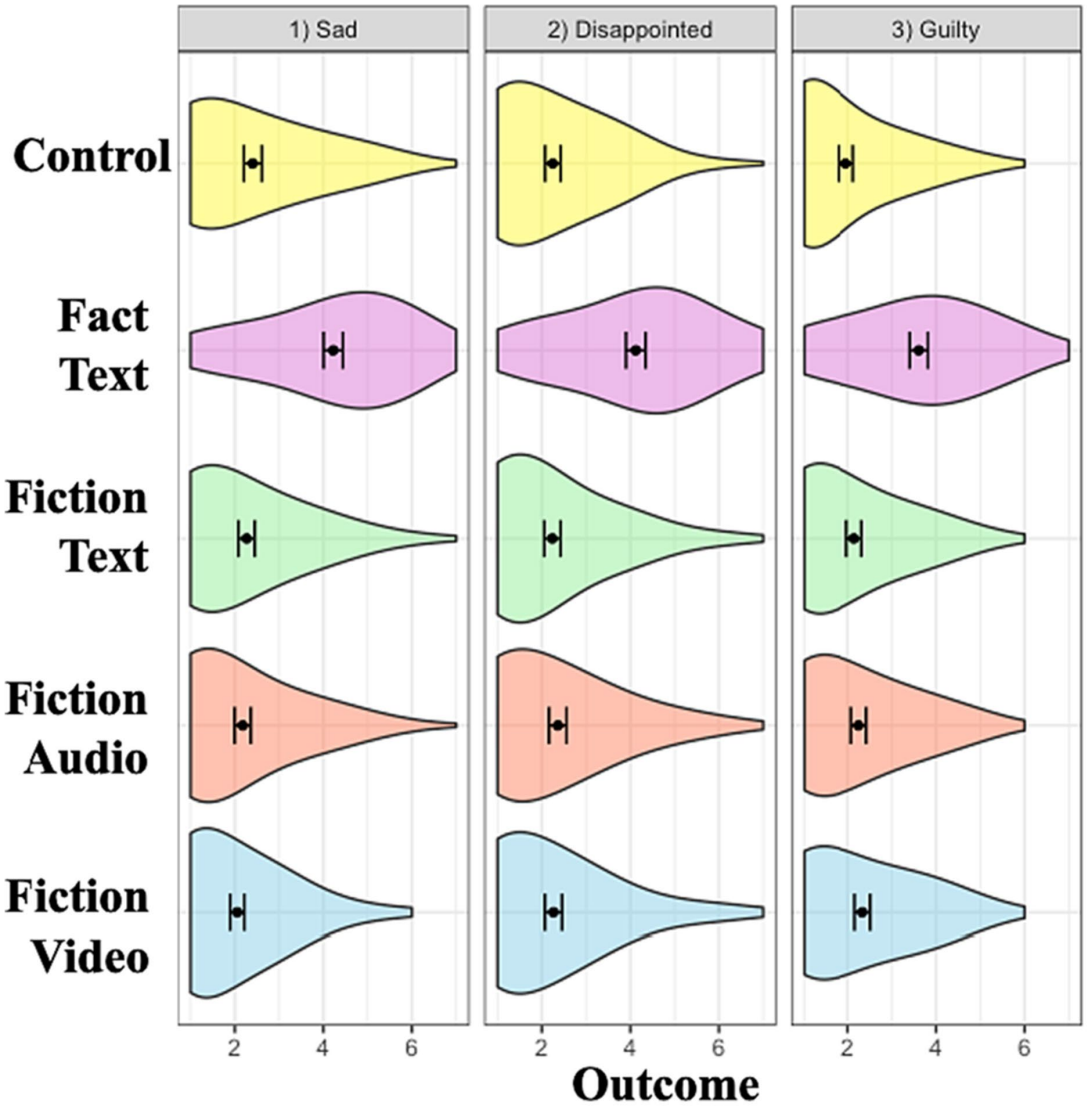
Conditional effects on negative valence emotions. Error bars represent 95% confidence interval of the means. Each emotion measured using a single item on a 7-point Likert scale.

Taken together, these findings indicate that the fiction video condition was more effective than other media in enhancing donations and positive valenced emotions. Interestingly, the audio and video conditions also increased guilt (Figures 2–5).

## 5 Discussion and conclusion

There is an urgent need to engage the public and persuade action on climate change. Aside from conventional efforts to communicate scientific information about the climate, there has been a proliferation of creative climate communications such as cli-fi. Cli-fi is communicated not only through textual means such as written novels, but also increasingly audio and audio-visual formats like podcasts and films. Yet the effects of these different modes on climate policy support and actions are unclear. The present study explores the effectiveness of scientific information and fiction-based climate communication on support for climate policy, individual and collective action intentions, and donations. In addition, it examines audience responses to cli-fi presented in textual, audio, and audio-visual communication media. This study adds causal evidence to existing work about the effectiveness of different types of content and media used in effective climate communication.

We found that cli-fi video stories (of people taking intentional climate action in the present) increased pro-environmental donations. This result is also partially in line with Morris et al. (2019), who also found that that exposure to personalized fiction stories can increase eco-donations. However, it also deviates from this past work as we find no differences in donations or intentions between the scientific information and fiction-text conditions [as in Morris et al. (2019)], or between fiction-audio and fiction-text [as in Riggs and Knobloch-Westerwick (2022)]. If exposure to any fiction condition increased donations, it is possible that the effect may have been driven by the narrative and story content itself. Since this was not the case, it is possible that the positive effect on donations is attributable to both the story content *and* its video presentation, rather than just the former. In line with this, the current study also replicates the finding from Sabherwal and Shreedhar (2022), who found that the same textual cli-fi narrative has no effect on donations compared to the control story. The result that cli-fi videos are more effective at increasing individual-level prosocial actions like donations, suggests that positive stories of people taking actions are effective when they are presented in an audio-visual format. More broadly, this result can speak to the power of personal climate stories as an effective form of climate communication (Markowitz et al., 2014; Howarth et al., 2020). Apart from personal stories of people being affected by climate change and extreme weather (e.g., see WaterAid’s campaign), our findings suggest that communicating personal stories of people trying to take action to mitigate climate change, such as changing diets can also be helpful to promote behavior change. Future work could explore how, when, and why such stories change behaviors: for example, do they change perceived pro-environmental social norms? does matching the character to the audience’s attributes (e.g., by age, gender, or ethnicity) increase effectiveness?

One reason that the fiction-video condition may have increased donations is because it stimulated positive emotions, such as happiness, hope and inspiration. Past research suggests that there can be a virtuous loop between happiness and prosocial behaviors, i.e., greater happiness increases prosocial actions such as volunteering and donations, and such actions in turn, further enhance positive feelings (Aknin et al., 2012; Park et al., 2017). Others note that generous behaviors, including toward the environment, are driven by the anticipation of a “warm glow” (or a positive affect arising from prosocial behavior; Andreoni, 1990; Taufik et al., 2015). We find that whereas reading scientific information about climate change enhanced negative emotions (sadness, disappointment, and guilt), watching a climate fiction video enhanced positive emotions. Both negative and positive affect are distinct pathways which can have downstream effects on support for climate policy and action. Therefore, research seeking to understand various tools of effective climate change communication need not limit itself to simply comparing different contents and formats of communication – instead, it can focus on the psychological mechanisms and boundary conditions that make a climate change communication effective. Communication strategies can enhance the positive and negative affect pathways we identity here such that, climate fiction can be designed to also target people’s positive, and scientific information their negative emotions. Although we consider affective responses as outcomes, there is much literature which explores how emotions are crucial predictors of risk perceptions, policy support, and technology acceptance. Therefore, it is possible, that repeated exposure to cli-fi such as ours can trigger a positive affect feedback loop – watching the story may enhance positive feelings (such as hope) that motivate pro-environmental action. Performing pro-environmental action may in-turn generate positive feelings (or warm glow), hence prompting further pro-environmental actions. In such a way, communication media that produce positive affect can induce sustained behavior change (Brosch, 2021). Such long-term effects of different forms of climate communication on emotions and behaviors is a promising area for future work.

That said, only the scientific information condition increased self-reported policy support. This result suggests that the effects of scientific information vs. fiction can depend on the outcome, and both have a place in the effective climate communication toolkit. When turning to the question of why we see these effects, the answer is not straightforward, as is evident from the prior discussion. For the sake of ecological validity, we designed our stimuli with many differences between the scientific information and fiction condition, including the ease of reading comprehension and content (although we controlled for word length). For example, in terms of the content, it is interesting to note that both the scientific information and fiction conditions showcased possible solutions but at the policy- and individual-level, respectively. Thus, if content drives the effect, it is possible to infer that exposure to scientific information about structural and systemic solutions bought about a shift in public attitudes congruent with this type of content. This suggests that to bring about system-wide policy changes, communicating scientific information remains essential. This may be especially in settings where political parties seek votes: for example, in Britain increased public concern about climate change was seen to be important for the development of the main parties’ climate policy preferences in the late 2000s (Carter and Jacobs, 2014).

### 5.1 Limitations and future directions

Future work on effective climate communication can expand the study design in various ways. Firstly, since this is a controlled online experimental survey study conducted in the United Kingdom, there is a debate about how these findings generalize to other populations and naturalistic settings. This study involved a large sample of British residents, including non-students. But it remains unclear how these results generalize to other political, geographical, and cultural contexts and populations. Future work could consider how the same cli-fi story could perform across different countries and within various sub-samples and socio-cultural groups within the same country.

Secondly, it is unclear if these effects on donations generalize to naturalistic settings when people face donation requests, such as while browsing the internet or during face-to-face interactions. To measure donations, we employed an incentivized modified dictator game with real monetary stakes. This approach aimed to reduce hypothetical response bias, which is a limitation of self-reported non-incentivized donation measures more commonly used in the literature (Carpenter et al., 2008; Lange, 2023). We also offered a diverse list of charities, to mitigate the impact of having a limited choice between charities (Carpenter et al., 2008). We chose to pay out a random subset of participants since previous research has shown that the effects are not significantly different when comparing a randomly selected subset of participants to paying all participants (Charness et al., 2016). We provided a windfall amount from which participants could make donations to ensure a minimum and fair payout to all participants for their time, regardless of their choices in the experiment. But it is possible that donations may have been lower if participants had to use their own earnings (Li et al., 2018). Indeed, there are several debates about how altruistic and pro-environmental behaviors elicited through incentivized experimental tasks generalize to behavior in real-world settings due to the many differences between these settings, including monetary stakes, the recipient, the presence of others, and other contextual factors (Franzen and Pointner, 2013; Galizzi and Navarro-Martinez, 2019; Lange, 2023). Future work should explore alternative methods, such as randomized controlled field trials in naturalistic settings [e.g., in Shreedhar (2021)], to examine how stories can causally impact charitable donation behavior in the real world.

Thirdly, this study only measured the immediate effects of communication messages. However, past research finds that the downstream effects of reading fiction might increase over time (Appel and Richter, 2007). A longitudinal study may, therefore, be more potent in evaluating the long-term effects of cli-fi (and scientific information) on environmental outcomes.

Fourthly, our experimental design focused on comparing different types of cli-fi story media (text, audio, and audio-visual) to either a textual control story or factual information. The scientific information in our study was only presented in text form. However, the way that the story is perceived and understood itself may depend on how the story and the medium interact. Future research can examine the effect of presenting scientific information in different, and potentially more esthetically appealing, media and narrative formats such as documentaries or TV series (e.g., Dahlstrom, 2014). It can also investigate whether such interactions between the message content and medium affect donations and other altruistic actions (Wong-Parodi and Feygina, 2021).

Finally, future work can also enhance the ecological validity of the climate fiction narrative. Since the cli-fi in the present study was devised by researchers to fit specific research questions, it may not be an ecologically valid representation of climate fiction written by authors. Furthermore, although the cli-fi was introduced as a “story” to participants, we did not formally assess whether they perceived it as fiction or a real account of the main character’s day. Therefore, future work should test the effect of communicating real-world cli-fi and assess the perceived fictionality of the story, on both altruistic behavior as well as other mechanisms such as narrative transportation.

## Data availability statement

The datasets presented in this study can be found in online repositories. The names of the repository/repositories and accession number(s) can be found in the article/Supplementary material.

## Ethics statement

The studies involving humans were approved by London School of Economics and Political Science ethics review board. The studies were conducted in accordance with the local legislation and institutional requirements. The participants provided their written informed consent to participate in this study.

## Author contributions

GS: conceptualization, methodology, investigation, resources, writing – original draft, writing – review and editing, supervision, project administration, and funding acquisition. AS: conceptualization, methodology, investigation, formal analysis, data curation, visualization, writing – original draft, writing – review and editing. RM: conceptualization, methodology, writing – review and editing. All authors contributed to the article and approved the submitted version.

## Footnotes

^1^ https://osf.io/kc96h/?view_only=3605345b565d459f864520eead18710d

^2^ https://osf.io/enavm/?view_only=c1ef0aa0b68e45fba5463022e38ab8dc

^3^Makaco Animation Studio.

^4^Oli Savage, Artistic Director, The Greenhouse.

^5^ https://osf.io/enavm/?view_only=c1ef0aa0b68e45fba5463022e38ab8dc

^6^ https://osf.io/kc96h/?view_only=3605345b565d459f864520eead18710d
