# Supplementary material for: Cli-fi videos can increase charitable donations: experimental evidence from the United Kingdom
Source: Front Psychol. 2024 Jan 19;14:1176077. doi: 10.3389/fpsyg.2023.1176077 (PMC10836214; doi:10.3389/fpsyg.2023.1176077)
Supplement: Supplementary file 1 [file Data_Sheet_1.PDF]

Supplementary Material

Cli-fi videos can increase charitable donations: Experimental evidence from the UK

Ganga Shreedhar [1*], Anandita Sabherwal[1,2], Ricardo Maldonado [1,3]

[1]Department of Psychological and Behavioural Sciences, London School of Economics and Political Science, London, United Kingdom of Great Britain and Northern Ireland

[2]Grantham Research Institute on Climate Change and the Environment, London School of Economics and Political Science, London, United Kingdom of Great Britain and Northern Ireland

[3]Cine70

*** Correspondence:**

Corresponding Author

g.s.shreedhar@lse.ac.uk

1 Supplementary Data

1.1 Sample Characteristics

The conditions were similarly distributed in terms of sample characteristics:

1.1.1 Gender

- The text condition comprised 107 males and 110 females
- The video condition comprised 109 males and 108 females
- The audio condition comprised 103 males and 108 females
- The control condition comprised 110 males and 98 females
- The informational appeal condition comprised 107 males and 114 females

1.1.2 Ethnicity

- The text condition comprised 194 White, 4 Black, African, Caribbean or Black British, 15 Asian or Asian British and 6 Mixed race identifying participants
- The video condition comprised 204 White, 2 Black, African, Caribbean or Black British, 10 Asian or Asian British, 3 Mixed race and 1 other ethnicity identifying participants
- The audio condition comprised 188 White, 5 Black, African, Caribbean or Black British, 12 Asian or Asian British, 5 Mixed race and 2 other ethnicity identifying participants
- The control condition comprised 189 White, 4 Black, African, Caribbean or Black British, 11 Asian or Asian British, 5 Mixed race and 3 other ethnicity identifying participants
- The informational appeal condition comprised 193 White, 10 Black, African, Caribbean or Black British, 12 Asian or Asian British, 3 Mixed race and 3 other ethnicity identifying participants

1.1.3 Political Party

- The text condition comprised 84 Labour, 46 Conservative, 13 Scottish National Party, 31 Liberal Democrat, 32 Green Party and 7 UK Independence Party supporters
- The video condition comprised 95 Labour, 50 Conservative, 8 Scottish National Party, 18 Liberal Democrat, 30 Green Party and 15 UK Independence Party supporters
- The audio condition comprised 80 Labour, 51 Conservative, 9 Scottish National Party, 26 Liberal Democrat, 35 Green Party and 7 UK Independence Party supporters
- The control condition comprised 88 Labour, 49 Conservative, 14 Scottish National Party, 20 Liberal Democrat, 25 Green Party and 11 UK Independence Party supporters

- The informational appeal condition comprised 109 Labour, 42 Conservative, 10 Scottish National Party, 25 Liberal Democrat, 21 Green Party and 8 UK Independence Party supporters

1.1.4 Brexit Support

- The text condition comprised 46 participants who supported the UK leaving the EU, 137 who supported the UK remaining within EU and 36 who neither supported nor opposed Brexit.
- The video condition comprised 56 participants who supported the UK leaving the EU, 126 who supported the UK remaining within EU and 38 who neither supported nor opposed Brexit.
- The audio condition comprised 47 participants who supported the UK leaving the EU, 127 who supported the UK remaining within EU and 39 who neither supported nor opposed Brexit.
- The control condition comprised 49 participants who supported the UK leaving the EU, 131 who supported the UK remaining within EU and 32 who neither supported nor opposed Brexit.
- The informational appeal condition comprised 44 participants who supported the UK leaving the EU, 140 who supported the UK remaining within EU and 37 who neither supported nor opposed Brexit.

1.1.5 Diet

The number of participants who had a meatless (were vegan or vegetarian) diet across conditions was:

- 25 participants in the text condition
- 21 participants in the video condition
- 21 participants in the audio condition
- 26 participants in the control condition
- 24 participants in the informational appeal condition

2 Supplementary Figures and Tables

2.1 Supplementary Figures

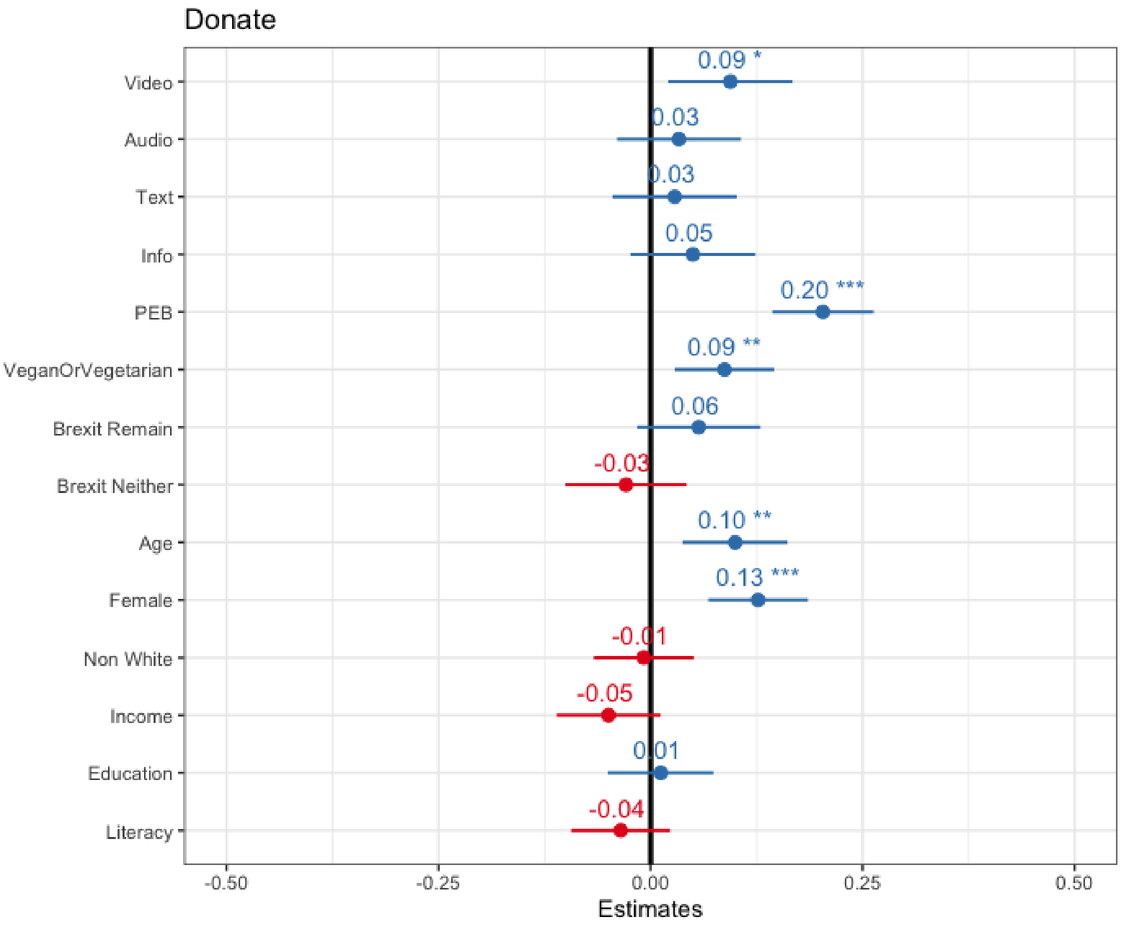

Supplementary Figure 1: Regression Model: Donation Amount Note: (i) * p<0.05, ** p<0.01, *** p<0.001; (iii) Estimates are standardized coefficients (iii) Error bars represent 95% confidence; (iv) Blue and red represent positive and negative effects respectively; (v) All outcomes measured using composites, on Likert scales of 1(not at all) to 7(extremely); (vi) Omitted categories: Control group, non-vegetarian/vegan diet, Brexit: leave, male, white; (vii) Scales for ordinal/numerical variables: PEB (Past environmental behavior)– 0-2 scale, Age– continuous numerical variable, Income– Likert scale of 1(less than £20,000) to 6 (more than £100,000), Education– Likert scale of 1(less than O level) to 10 (Doctorate or other professional degree), Literacy– Likert scale of 1 (no proficiency) to 6 (native/bilingual proficiency)

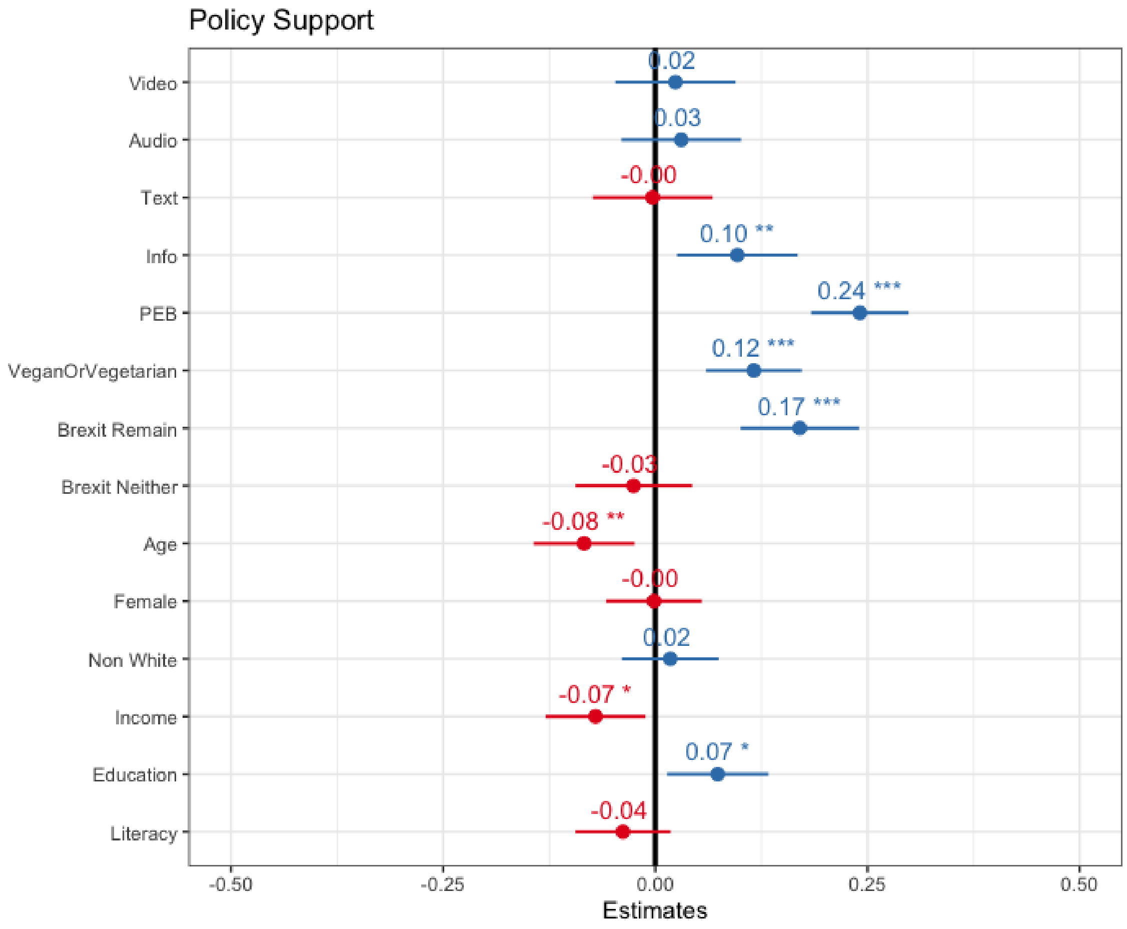

Supplementary Figure 2: Regression Model: Policy Support Note: (i) * $p<0.05$, ** $p<0.01$, *** $p<0.001$; (iii) Estimates are standardized coefficients (iii) Error bars represent 95% confidence; (iv) Blue and red represent positive and negative effects respectively; (v) All outcomes measured using composites, on Likert scales of 1(not at all) to 7(extremely); (vi) Omitted categories: Control group, non-vegetarian/vegan diet, Brexit: leave, male, white; (vii) Scales for ordinal/numerical variables: PEB (Past environmental behavior)– 0-2 scale, Age– continuous numerical variable, Income– Likert scale of 1(less than £20,000) to 6 (more than £100,000), Education– Likert scale of 1(less than O level) to 10 (Doctorate or other professional degree), Literacy– Likert scale of 1 (no proficiency) to 6 (native/bilingual proficiency)

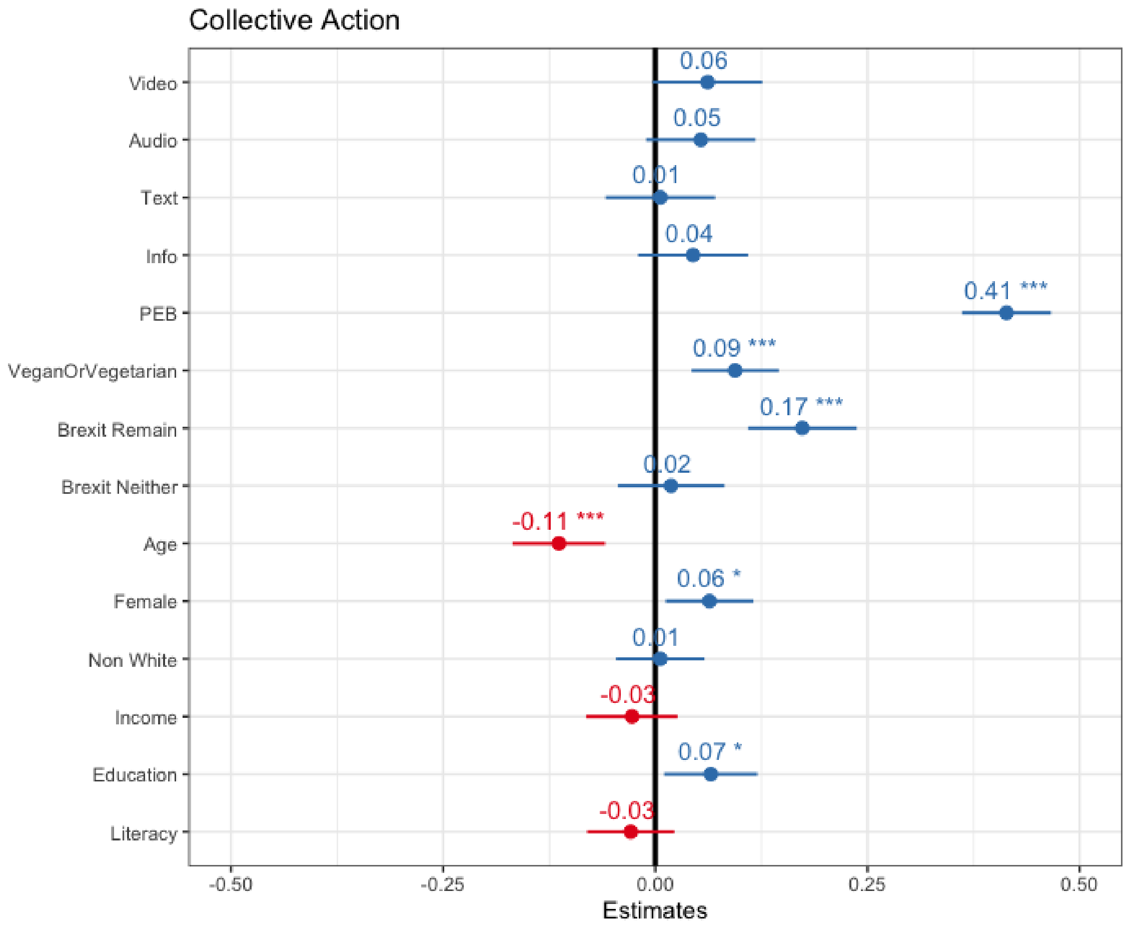

Supplementary Figure 3: Regression Model: Collective Action Intentions Note: (i) * *p*<0.05, ** *p*<0.01, *** *p*<0.001; (iii) Estimates are standardized coefficients (iii) Error bars represent 95% confidence; (iv) Blue and red represent positive and negative effects respectively; (v) All outcomes measured using composites, on Likert scales of 1(not at all) to 7(extremely); (vi) Omitted categories: Control group, non-vegetarian/vegan diet, Brexit: leave, male, white; (vii) Scales for ordinal/numerical variables: PEB (Past environmental behavior)– 0-2 scale, Age– continuous numerical variable, Income– Likert scale of 1(less than £20,000) to 6 (more than £100,000), Education– Likert scale of 1(less than O level) to 10 (Doctorate or other professional degree), Literacy– Likert scale of 1 (no proficiency) to 6 (native/bilingual proficiency)

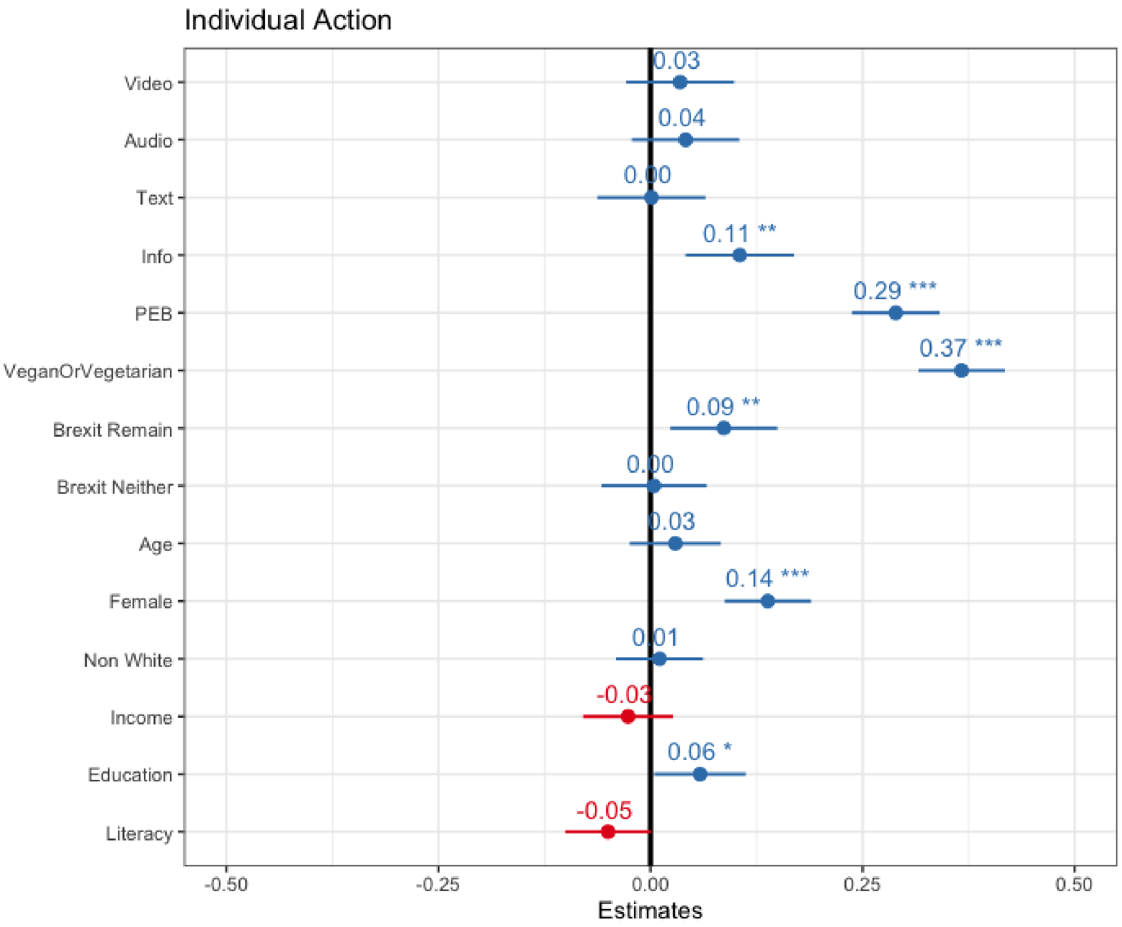

Supplementary Figure 4: Regression Model: Individual Action Intentions Note: (i) * $p<0.05$, ** $p<0.01$, *** $p<0.001$; (iii) Estimates are standardized coefficients (iii) Error bars represent 95% confidence; (iv) Blue and red represent positive and negative effects respectively; (v) All outcomes measured using composites, on Likert scales of 1(not at all) to 7(extremely); (vi) Omitted categories: Control group, non-vegetarian/vegan diet, Brexit: leave, male, white; (vii) Scales for ordinal/numerical variables: PEB (Past environmental behavior)– 0-2 scale, Age– continuous numerical variable, Income– Likert scale of 1(less than £20,000) to 6 (more than £100,000), Education– Likert scale of 1(less than O level) to 10 (Doctorate or other professional degree), Literacy– Likert scale of 1 (no proficiency) to 6 (native/bilingual proficiency)

2.2 Supplementary Tables

Supplementary Table 1: Descriptive statistics of socio-demographic features

Variable	Sample M (SD)	Text M(SD)	Video M(SD)	Audio M(SD)	Control M(SD)	Informational Appeal M(SD)	Difference across conditions (ANOVA *p* value)
Age	40.50 (12.91)	40.46 (13.06)	39.28 (12.46)	41.65 (12.58)	41.02 (13.76)	40.14 (12.67)	0.38
Past Pro-Environmental Behaviour	0.67 (0.77)	0.74 (0.79)	0.73 (0.79)	0.63 (0.77)	0.66 (0.77)	0.59 (0.72)	0.22
Covid worry	3.51 (1.66)	3.78 (1.69)	3.42 (1.69)	3.46 (1.71)	3.45 (1.61)	3.43 (1.58)	0.11
Political Ideology	3.44 (0.88)	3.42 (0.88)	3.40 (0.87)	3.49 (0.88)	3.48 (0.93)	3.42 (0.86)	0.74
Income	1.94 (1.02)	2.02 (1.05)	1.93 (1.08)	1.93 (0.96)	1.94 (1.05)	1.89 (0.95)	0.72
Education	6.31 (2.68)	6.45 (2.56)	6.43 (2.60)	6.21 (2.71)	6.42 (2.76)	6.04 (2.77)	0.41
Literacy	5.91 (0.34)	5.92 (0.31)	5.89 (0.42)	5.92 (0.31)	5.86 (0.37)	5.93 (0.27)	0.20

Note. Table summarizes demographic information for the sample and per condition. We conduct one-way ANOVAs to show that participants' demographic characteristics did not differ significantly across conditions. Age was measured in years; Past Pro-environmental behavior measured whether participants had donated to an environmental charity and/or joined a protest in the past on a scale of 0 (neither) to 2 (both); Covid worry was measured on a Likert scale of 1 (Not at all) to 7 (Very much); Political ideology was measured on a Likert scale of 1 (Very conservative) to 5 (Very liberal); Income was measured on a Likert scale of 1 (£20,000 or less annual income) to 6 (more than £100000 annual income); Education was measured on a Likert scale of 1 (Less than O level) to 10 (Doctorate or other professional qualification) and Literacy was measured on a Likert scale of 1 (No English language proficiency) to 6 (Native/Bilingual English language proficiency).

Supplementary Table 2: Descriptive statistics of variables of interest

Variable	Sample M (SD)	Text M(SD)	Video M(SD)	Audio M(SD)	Control M(SD)	Informational Appeal M(SD)
Donation amount	0.42 (0.35)	0.42 (0.35)	0.47 (0.35)	0.41 (0.34)	0.39 (0.34)	0.42 (0.36)
Policy support	4.55 (1.59)	4.45 (1.58)	4.53 (1.59)	4.51 (1.62)	4.54 (1.64)	4.78 (1.49)
Collective action intentions	3.40 (1.61)	3.53 (1.70)	3.54 (1.61)	3.42 (1.59)	3.29 (168)	3.38 (1.48)
Individual action intentions	3.62 (1.67)	3.52 (1.73)	3.60 (1.67)	3.60 (1.67)	3.50 (1.70)	3.85 (1.58)
ECAS	3.83 (1.24)	3.67 (1.24)	4.03 (1.26)	3.74 (1.19)	3.88 (1.21)	3.86 (1.26)
Transportation	4.02 (1.21)	4.03 (1.27)	4.06 (1.20)	4.08 (1.19)	3.85 (1.20)	4.05 (1.18)
Happy	3.32 (1.47)	3.37 (1.50)	3.86 (1.42)	3.51 (1.43)	3.43 (1.39)	2.48 (1.22)
Surprised	2.79 (1.43)	2.80 (1.48)	2.88 (1.43)	2.72 (1.36)	2.83 (1.38)	2.69 (1.49)
Hopeful	3.56 (1.54)	3.61 (1.65)	4.01 (1.58)	3.68 (1.53)	3.23 (1.43)	3.28 (1.36)
Inspired	3.58 (1.58)	3.52 (1.68)	3.95 (1.55)	3.51 (1.56)	3.37 (1.63)	3.56 (1.44)
Sad	2.63 (1.64)	2.26 (1.39)	2.05 (1.21)	2.18 (1.35)	2.41 (1.49)	4.22 (1.64)
Guilty	2.46 (1.46)	2.14 (1.27)	2.34 (1.34)	2.25 (1.29)	1.95 (1.22)	3.61 (1.56)
Disappointed	2.65 (1.63)	2.34 (1.38)	2.26 (1.45)	2.36 (1.46)	2.25 (1.29)	4.12 (1.66)

Supplementary Table 3: Intercorrelations between variables of interest

	A.	B.	C.	D.	E.	F.	G.	H.	I.	J.	K.	L.
A. Collective Action Intention	1											
B. Individual Action Intention	0.6***	1										
C. Policy Support	0.62***	0.55***	1									
D. Donation Amount	0.32***	0.3***	0.31***	1								
E. ECAS	0.45***	0.44***	0.38***	0.16***	1							
F. Transportation	0.42***	0.35***	0.33***	0.18***	0.31***	1						
G. Happy	0.26***	0.19***	0.18***	0.18***	0.25***	0.42***	1					
H. Surprised	0.18***	0.11***	0.1***	0.11***	0.14***	0.33***	0.36***	1				
I. Hopeful	0.34***	0.28***	0.24***	0.22***	0.31***	0.5***	0.69***	0.42***	1			
J. Inspired	0.35***	0.3***	0.3***	0.22***	0.28***	0.56***	0.65***	0.4***	0.74***	1		
K. Sad	0.14***	0.16***	0.16***	0.05 $^{+}$	0.07*	0.21***	-0.17***	0.19***	0.03	0.09**	1	
L. Disappointed	0.06*	0.09**	0.06*	0.03	0.04	0.08**	-0.22***	0.17***	-0.05 $^{+}$	-0.03	0.68***	1
M. Guilty	0.23***	0.17***	0.24***	0.12***	0.11***	0.34***	0.03	0.27***	0.2***	0.3***	0.56***	0.45***

Note: (i) $^{+}$ p<0.10, * p<0.05, ** p<0.01, *** p<0.001; (iii) Estimates are Pearson's r correlations coefficients of bivariate correlational analyses.

Supplementary Table 4: Main Effect of Treatment (all 5 conditions) on Variables of Interest

**Variable**	**ANOVA**
Donation	*p>0.10*
Policy Support	*p>0.10*
Individual Action Intentions	*p>0.10*
ECAS	$F_{(4,1080)}=2.90$, $p = 0.02$, $\eta_p^2=0.01$
Transportation	*p>0.10*
Happiness	$F_{(4,1080)} =29.82$, $p < 0.001$, $\eta_p^2=0.10$
Hope	$F_{(4,1080)}=9.78$, $p < 0.001$, $\eta_p^2=0.03$
Inspiration	$F_{(4,1080)}= 10.42$, $p = 0.002$, $\eta_p^2=0.04$
Surprise	$p>0.10$
Sadness	$F_{(4,1080)}=88.59$, $p < 0.001$, $\eta_p^2=0.25$.
Guilt	$F_{(4,1080)} = 53.11$, $p < 0.001$, $\eta_p^2=0.16$
Disappointment	$F_{(4,1080)} = 70.98$, $p < 0.001$, $\eta_p^2=0.21$

3 Deviation from Pre-registration

3.1 Hypotheses

In the pre-registration, we hypothesize that the audio-visual (video) condition will have the largest positive impact on pro-environmental outcomes (H1) and narrative transportation (H2) compared to all other conditions. However, upon conducting a more thorough review of the literature, we realized that the fact condition (adapted from IPCC) was qualitatively different from the video and audio fiction conditions in two ways– its format (text vs. audio vs video) and its content (fictional vs. factual). Therefore, a comparison of the fiction video (or audio) and fact conditions would not inform either of our research questions.

Therefore, our first and second hypotheses can be qualified as– the video condition will have the largest positive impact on pro-environmental outcomes (H1) a narrative transportation (H2) compared to the fiction text, fiction audio, and control conditions.

Moreover, although we did not have (and did not pre-register) a directional hypothesis for this, we also examined the difference in environmental outcomes, narrative transportation and emotions between the fact text, fiction text and control conditions.

3.2 Analyses

First, we pre-registered that, "The primary analyses will be one-way ANOVAs and planned pairwise contrasts between conditions on the outcome variables mentioned above." However, as noted above, we recognize that the fact text condition is not comparable to the fiction audio and video conditions. Therefore, we conducted two sets of one-way ANOVAs followed by pairwise contrast. These are outlined below:

- **To compare the effectiveness of fact vs fiction: ANOVA of treatment (fact text, fiction text and control) on variables of interest followed by pairwise contrasts**
- **To compare the effectiveness of media: ANOVA of treatment (fiction text, fiction audio, fiction video, and control) on variables of interest followed by pairwise contrasts**

We also report the original pre-registered analysis in the supplement (See Supplementary Table 4). Second, we pre-registered that we will conduct serial mediation o test Hypothesis 3 but we did not conduct this analysis because there was no main effect of condition (video vs audio vs text fiction vs control) on any of the environmental actions, therefore making the serial mediation analysis to assess mechanisms of the effect void.
